# Central vein sign and diffusion MRI differentiate microstructural features within white matter lesions of multiple sclerosis patients with comorbidities

**DOI:** 10.3389/fneur.2023.1084661

**Published:** 2023-03-08

**Authors:** Caterina Lapucci, Francesco Tazza, Silvia Rebella, Giacomo Boffa, Elvira Sbragia, Nicolò Bruschi, Elisabetta Mancuso, Nicola Mavilio, Alessio Signori, Luca Roccatagliata, Maria Cellerino, Simona Schiavi, Matilde Inglese

**Affiliations:** ^1^HNSR, IRRCS Ospedale Policlinico San Martino, Genoa, Italy; ^2^Department of Neuroscience, Rehabilitation, Ophthalmology, Genetics, Maternal and Child Health (DINOGMI), University of Genoa, Genoa, Italy; ^3^University of Genoa, Genoa, Italy; ^4^Department of Neuroradiology, IRCCS Ospedale Policlinico San Martino, Genoa, Italy; ^5^Department of Health Sciences (DISSAL), University of Genoa, Genoa, Italy; ^6^IRCCS Ospedale Policlinico San Martino IRCCS, Genoa, Italy

**Keywords:** multiple sclerosis, comorbidities, MRI, central vein sign, diffusion

## Abstract

**Introduction:**

The Central Vein Sign (CVS) has been suggested as a potential biomarker to improve diagnostic specificity in multiple sclerosis (MS). Nevertheless, the impact of comorbidities on CVS performance has been poorly investigated so far. Despite the similar features shared by MS, migraine and Small Vessel Disease (SVD) at T2-weighted conventional MRI sequences, *ex-vivo* studies demonstrated their heterogeneous histopathological substrates. If in MS, inflammation, primitive demyelination and axonal loss coexist, in SVD demyelination is secondary to ischemic microangiopathy, while the contemporary presence of inflammatory and ischemic processes has been suggested in migraine. The aims of this study were to investigate the impact of comorbidities (risk factors for SVD and migraine) on the global and subregional assessment of the CVS in a large cohort of MS patients and to apply the Spherical Mean Technique (SMT) diffusion model to evaluate whether perivenular and non-perivenular lesions show distinctive microstructural features.

**Methods:**

120 MS patients stratified into 4 Age Groups performed 3T brain MRI. WM lesions were classified in “perivenular” and “non-perivenular” by visual inspection of FLAIR^*^ images; mean values of SMT metrics, indirect estimators of inflammation, demyelination and fiber disruption (EXTRAMD: extraneurite mean diffusivity, EXTRATRANS: extraneurite transverse diffusivity and INTRA: intraneurite signal fraction, respectively) were extracted.

**Results:**

Of the 5303 lesions selected for the CVS assessment, 68.7% were perivenular. Significant differences were found between perivenular and non-perivenular lesion volume in the whole brain (*p* < 0.001) and between perivenular and non-perivenular lesion volume and number in all the four subregions (*p* < 0.001 for all). The percentage of perivenular lesions decreased from youngest to oldest patients (79.7%–57.7%), with the deep/subcortical WM of oldest patients as the only subregion where the number of non-perivenular was higher than the number of perivenular lesions. Older age and migraine were independent predictors of a higher percentage of non-perivenular lesions (*p* < 0.001 and *p* = 0.013 respectively). Whole brain perivenular lesions showed higher inflammation, demyelination and fiber disruption than non perivenular lesions (*p* = 0.001, *p* = 0.001 and *p* = 0.02 for EXTRAMD, EXTRATRANS and INTRA respectively). Similar findings were found in the deep/subcortical WM (*p* = 0.001 for all). Compared to non-perivenular lesions, (i) perivenular lesions located in periventricular areas showed a more severe fiber disruption (*p* = 0.001), (ii) perivenular lesions located in juxtacortical and infratentorial regions exhibited a higher degree of inflammation (*p* = 0.01 and *p* = 0.05 respectively) and (iii) perivenular lesions located in infratentorial areas showed a higher degree of demyelination (*p* = 0.04).

**Discussion:**

Age and migraine have a relevant impact in reducing the percentage of perivenular lesions, particularly in the deep/subcortical WM. SMT may differentiate perivenular lesions, characterized by higher inflammation, demyelination and fiber disruption, from non perivenular lesions, where these pathological processes seemed to be less pronounced. The development of new non-perivenular lesions, especially in the deep/subcortical WM of older patients, should be considered a “red flag” for a different -other than MS- pathophysiology.

## Introduction

Multiple sclerosis (MS) is an inflammatory disease of the central nervous system (CNS) characterized by a relapsing or progressing clinical course. Although focal hyperintensities on T2-weighted magnetic resonance imaging (MRI) detected within the brain and spinal cord represent the radiological hallmarks of the disease (1), they lack histopathological specificity and may hide heterogeneous pathological substrates.

The perivenular location of MS lesions has been known for more than a century. From a histopathological point of view, MS lesions are characterized by cellular infiltrates that rise around small-to-medium-sized parenchymal venules (2), the so-called “perivascular cuffs”, mainly characterized by mononuclear cells that enter CNS by damaging the blood-brain barrier (BBB) as waves of inflammatory invasion (3). The essential transition from the histopathological evidence to the “*in vivo*” demonstration of the presence of a central venule within MS lesions has been made possible by advanced gradient-echo MRI techniques (4, 5). Thus, this “Central Vein Sign” (CVS) has been suggested as a potential biomarker to improve diagnostic specificity in MS (6–8).

Nevertheless, the presence of cardiovascular comorbidities, which are particularly frequent in older patients with progressive MS, introduces an extra challenge in the conventional radiological setting, where advanced and specific MRI biomarkers may be needed to distinguish whether a new T2-weighted lesion is due to MS or age-related comorbidities. The prevalence of small vessel disease (SVD)-related white matter (WM) hyperintensities increases from approximately 5% for people aged 50 years to nearly 100% for people aged 90 years (9). In addition to age, arterial hypertension (HT) (10), current and former smoking, and diabetes mellitus (11) are considered modifiable risk factors (RFs) for SVD.

However, data about the impact of age and, more generically, of other RFs for SVD on CVS performance in patients with MS are still scarce. In a recent study, performed on a relatively small cohort of patients with MS, the percentage of CVS+ (from now on “%CVS+”) lesions significantly decreased in older and hypertensive patients with MS (12).

Besides SVD, migraine is a frequent comorbidity in patients with MS (13). It is well-known that WM T2-weighted hyperintensities are frequently detected in patients with migraine and persist over time (14), with the deep/subcortical WM of the frontal lobes typically involved (15). Although previous studies explored how to differentiate MS from migraine by using MRI (16) and how migraine may be associated with a more symptomatic MS course (17), the impact of migraine as a comorbidity in MS diagnosis and radiological monitoring has not yet been deeply investigated.

Despite the similar features shared by MS, migraine, and SVD-related WM T2-weighted hyperintensities on conventional MRI, *ex vivo* studies showed the heterogeneity of the underlying histopathological substrates (18, 19). Nevertheless, the microstructural features differentiating WM lesions due to MS from WM lesions due to comorbidities have not yet been investigated by using *in vivo* MRI.

To overcome the limited pathological specificity of conventional MRI, several advanced MRI techniques have been developed and applied to characterize microstructural alterations due to tissue disruptions caused by MS (20, 21). Among all the proposed multicompartment models, the spherical mean technique (SMT) has been successfully applied to characterize the brain (22) and the spinal cord (23) of patients with MS. Nevertheless, to the best of our knowledge, whether CVS+ lesions show distinctive microstructural features compared to CVS− lesions has not yet been investigated.

Therefore, the aims of our study were a) to investigate the impact of risk factors for SVD and migraine on the global and subregional brain CVS assessment in a large cohort of patients with MS as a whole and stratified according to age; b) to investigate the pathological substrate of CVS+ and CVS– lesions using advanced diffusion metrics (SMT); and c) to determine whether the use of SMT-derived metrics can differentiate perivenular lesions, typical of MS, from no perivenular lesions, possibly associated with different pathophysiological mechanisms related to comorbidities.

## Materials and methods

### Subjects

In this prospective study, 120 patients with a diagnosis of MS (24) [84 with relapsing-remitting (RRMS), 36 with progressive (Primary Progressive, PP and Secondary Progressive, SP, from now on “PMS”) disease course (25)] were consecutively enrolled between January 2019 and September 2020 at the Department of Neuroscience, Rehabilitation, Ophthalmology, Genetics, Maternal and Child Health (University of Genoa). Inclusion criteria were as follows: (I) age >18 years and (II) MS diagnosis according to revisions of McDonald's criteria (24). Exclusion criteria were as follows: (i) absence of capability to sign the informed consent and (ii) suboptimal MRI quality.

Moreover, we stratified the included subjects as follows: (i) Group 1: 18–30 years (*n* = 30); (ii) Group 2: 31–44 years (*n* = 30); (iii) Group 3: 45–55 years (*n* = 30); (iv) Group 4: 56–77 years (*n* = 30).

All patients underwent neurological examination with the assessment of the Expanded Disability Status Scale (EDSS). In addition, the following RFs for SVD were recorded: body mass index (BMI; measured as a weight-to-height ratio, cut-off ≥25 kg/m^2^), smoking (at the time of MRI examination or in the past), diagnosis of HT (at the time of MRI examination or in the past) and its medications, diabetes or glucose intolerance (at the time of MRI examination or in the past) and its medications, and hypercholesterolemia (at the time of MRI examination or in the past) and its medications. The cumulative number of RFs was calculated for each patient. Furthermore, the presence of migraine (or history of migraine) with or without aura (from now on simply “migraine”) was also recorded.

### MRI acquisition

All patients underwent MRI on a 3T Siemens MAGNETOM Prisma (Siemens Healthcare, Erlangen, Germany) with a 64-channel head and neck coil.

The MRI protocol included (i) 3D sagittal T2-FLAIR (repetition time/inversion time/echo time (TR/TI/TE): 5,000/1,800 ms/393 ms; resolution 0.4 × 0.4 × 1 mm^3^); (ii) 3D sagittal T1 MPRAGE (TR/TI/TE: 2300 ms/919 ms/2.96 ms; resolution 1 × 1 × 1 mm^3^) before and after intravenous contrast injection of 10 ml of 0.5 mmol/ml gadoteric acid contrast agent; (iii) twice-refocused spin echo echo-planar imaging sequence for multi-shell diffusion-weighted images (TR/TE: 4,500 /75 ms; 107 diffusion directions distributed in 5 shells with b-value up to 3,000 s/mm^2^ plus 7 non weighted images acquired with both anterior-posterior and posterior-anterior phase encoding directions; spatial resolution 1.8 × 1.8 × 1.8 mm^3^); (iv) 3D sagittal segmented echo-planar imaging (EPI) providing T2^*^ magnitude and phase contrasts (TR/TE: 64 ms/35 ms; resolution 0.65 × 0.65 × 0.65 mm^3^) after intravenous contrast injection of 10 ml of 0.5 mmol/ml gadoteric acid contrast agent.

### Lesion segmentation and CVS assessment

Central vein sign assessment was performed on FLAIR^*^ images obtained by rigid co-registration (26) and voxel-wise multiplication of the high-resolution 3D T2^*^ EPI and the 3D T2-FLAIR, as previously described (27).

FLAIR^*^ images were reformatted in the axial plane maintaining the native section thickness of 0.65 mm to improve the visualization of vessels within MS lesions and were used for the assessment of the presence of the CVS. For each patient, brain WM matter lesions were selected for the assessment of CVS according to NAIMS guideline (28). The presence or absence of the CVS (CVS+ lesions or “perivenular” and CVS− lesions or “non-perivenular”, respectively) was blindly and independently evaluated by two assessors (neurologists with expertise in neuroimaging of MS), according to the NAIMS guidelines (28). In the case of disagreement between assessors, lesions were reviewed by a third assessor (with great expertise in neuroimaging) and a consensus was reached. Gadolinium enhancing lesions were excluded from the analysis to avoid the possible contamination of FLAIR^*^ images due to the leakage of contrast agent within lesions with evidence of BBB disruption. Then, selected CVS+ and CVS− lesions were manually segmented on native FLAIR^*^ images using Jim software (Jim 7.0, Xinapse System; http://www.xinapse.com), creating CVS+ and CVS− lesion masks, respectively.

In addition, patients with MS were classified into “perivenular positive” vs. “perivenular negative” according to the previously proposed criteria: the 40% CVS proportion-based diagnostic thresholds (29–31), the “6-lesion rule” (8), and the “3-lesion rule” (32).

An in-house algorithm based on priors about tissues segmentation was used to automatically subdivide CVS+ and CVS− lesions according to their location: (i) deep/subcortical WM, (ii) periventricular, (iii) juxtacortical, and (iv) infratentorial. To avoid mislabelling, a quality check on the resulting classification was then made by a neurologist with more than 5 years of experience.

Finally, whole brain and subregion-specific CVS+ and CVS− lesion masks were registered on T1- weighted images using the automated FMRIB's Linear Image Registration Tool (FLIRT) with boundary-based registration (33).

### Diffusion processing

Diffusion MR images were first denoised using the Marchenko-Pastur principal component analysis algorithm (34) available in MRtrix3 (35). Then they were corrected for movement artifacts and susceptibility induced distortions using eddy and top-up commands from FMRIB Software Library (FSL) (36–39). As the last step of pre-processing, we also performed B1 field inhomogeneity correction to all the dMRI volumes (40). To compute the microstructural maps derived from the SMT model, we used the open-source code available at (https://github.com/ekaden/smt). To register the different lesion masks on the SMT maps, first, the diffusion weighted images were registered on T1-weighted images using FLIRT with boundary-based registration (33), then the resulting transformations were inverted and applied to the lesion masks to register them in the diffusion weighted image space. Similar to the study by Inglese et al. (41), to compensate for the variable partial volume effects caused by the different resolutions between the images, only lesions larger than three voxels after registration on diffusion space were included in the final data analysis. All the registrations were visually checked by a trained professional with more than 5 years of experience in neuroimaging. Finally, we extracted the mean values inside each type of lesions of the following SMT microstructural maps, namely intraneurite signal fraction (INTRA), extraneurite transverse diffusivity (EXTRATRANS), and extraneurite mean diffusivity (EXTRAMD), that describe the fraction of signal coming from the intra-axonal compartment as well as the properties of the anisotropic extraneurite compartment *via* its transverse microscopic diffusivity and mean diffusion outside the axons, respectively (42, 43).

### Statistical analysis

Results were reported as mean with standard deviation (SD) or median with range. Differences in lesion volume and lesion location frequencies were compared between CVS+ and CVS− using a generalized estimating equation (GEE) model to take into account multiple lesions from the same patients. The association of demographic and clinical characteristics of patients on the percentage of CVS lesions was assessed using the Mann-Whitney test for binary variables or the Kruskal-Wallis test for categorical variables. Spearman's rank correlation was used for continuous characteristics such as age, disease duration, and BMI. All significant (*p* < 0.05) characteristics at the univariable analyses were included in a multivariable linear regression model. Single lesions microstructural metrics comparisons between CVS+ and CVS− and according to age groups were performed using the GEE model for the same reasons reported above. The mean and SD of each microstructural metric were estimated from a multivariable GEE model also including age, gender, and MS type. EDSS scores were correlated with lesional and normal appearing white matter (NAWM) SMT-derived metrics by using Spearman's test. *P*-values were adjusted for multiple comparisons using the false-discovery rate (FDR) approach. Stata (v.16; Statacorp) was used for the computation.

Approval for this study was received from the Local Ethic Committee of the IRCCS Ospedale Policlinico San Martino (Genoa), and written informed consent was obtained from all subjects.

### Data availability

The 3T brain MRI images used were obtained from the IRCCS Ospedale Policlinico San Martino of Genoa and could be made available from the corresponding author upon reasonable request.

## Results

### Demographic and clinical data

In our cohort, 66 patients with MS were women (55%), the mean (±SD) age was 43.8 ± 14.4 years, and the mean disease duration was 13.4 ± 10.6 years. A more detailed summary of the demographic and clinical features of the enrolled subjects is reported in Table 1. No differences were present in terms of gender distribution. Disease duration was different between age Group 4 vs. age Group 1 and age Group 2 (*p* < 0.001 for both, >in age Group 4) and between age Group 3 vs. age Group 1 (*p* = 0.001, >in age Group 3) and age Group 2 (*p* < 0.001, >in age Group 3). No differences in disease duration were present between age Group 1 vs. age Group 2 and age Group 3 vs. age Group 4. MS phenotype was different between age Group 1 vs. Age Group 3 and age Group 4 [RRMS >PMS, *p* < 0.001 for both] and between age Group 2 vs. age Group 4 (RRMS>PMS, *p* = 0.002). HT was more prevalent in age Group 4 vs. age Group 3 (*p* = 0.04), age Group 2, and age Group 1 (*p* < 0.001 for both). A difference in the prevalence of hypercholesterolemia was observed between age Group 4 vs. age Group 1 (*p* = 0.021). No differences in terms of prevalence of migraine, smoke, diabetes or glucose intolerance were observed among age groups.

**Table 1:** Baseline demographic and clinical characteristics.

**Demographic and MS clinical data**	
Patients, *n*	120
Female, %	55
Age, years, mean (*SD)*	43.8 (14.4)
EDSS score, median (*range*)	2 (1–7)
**MS phenotype**, ***n*** **(*****%*****)**	
RRMS	84 (70)
SPMS	21 (17.5)
PPMS	15 (12.5)
Disease duration, years, mean (*SD*)	13.4 (10.6)
**Comorbidities clinical data**	
Age Groups, *n*	4
Age Group 1, *n*. patients (range, years)	30 (18–30 years)
Age Group 2, *n*. patients (range, years)	30 (31–44 years)
Age Group 3, *n*. patients (range, years)	30 (45–55 years)
Age Group 4, *n*. patients (range, years)	30 (56–77 years)
HT, *n* (%)	17 (*14.2*)
Diabetes or glucose intolerance, *n* (%)	2 (1.7)
Smoke, *n* (%)	63 (52.5)
BMI ≥25 kg/m^2^, *n* (%)	10 (8.3)
Hypercholesterolemia, *n* (%)	21 (17.5)
Cumulative number of RFs for SVD, median (*range*)	1 (0–4)
Migraine, *n* (%)	34 (28.3)
**Demographic and clinical features according to age Groups** ^a^	
Disease duration	1 vs. 4 **(*****p*** **<** **0.001)**
	2 vs. 4 **(*****p*** **<** **0.001)**
	1 vs. 3 **(*****p*** **=** **0.001)**
	2 vs. 3 **(*****p*** **<** **0.001)**
	(> in age Group 4 and age Group 3)
MS phenotype^*^	1 vs. 3 **(*****p*** **<** **0.001)**
	1 vs. 4 **(*****p*** **<** **0.001)**
	2 vs. 4 **(*****p*** **=** **0.002)**
	(RRMS > PMS in age Group 1)
HT	1 vs. 4 **(*****p*** **<** **0.001)**
	2 vs. 4 **(*****p*** **<** **0.001)**
	3 vs. 4 **(*****p*** **=** **0.04)**
	(>HT in age Group 4)
Hypercholesterolemia	1 vs. 4 **(*****p*** **=** **0.021)**
	(>hypercholesterolemia in age Group 4)

EDSS, Expanded Disability Status Scale; RRMS, Relapsing Remitting Multiple Sclerosis; SPMS, Secondary Progressive Multiple Sclerosis; PPMS, Primary Progressive Multiple Sclerosis; HT, arterial hypertension; BMI, Body Mass Index; RFs, risk factors; SVD, Small Vessel Disease.

^a^Only significant comparisons among age groups were reported.

^*^All PPMS patients were included in age Groups 3 and 4.

### CVS assessment: Global data and inter-assessor agreement

A total of 7,445 brain WM lesions were analyzed with a median of 27.3 (range: 4–51) lesions per patient. Among the 7,445 lesions, 5,303 (71.2%) were selected for CVS assessment. Of the 5,303 lesions, 3,645 (68.7%) were CVS+. The median frequency of CVS+ lesions per patient was 73.5% (range: 27.7–100%). The inter-assessor agreement for the percentage of CVS+ lesions was “substantial/good” with a Cohen's κ of 0.7 and an agreement of 89%.

Lesion volume was different between CVS+ and CVS– lesions (median = 1,292 mm3, range: 26–7,969 mm3 vs. 224 mm3, range: 17–1,713 mm3, respectively; *p* < 0.001). CVS+ lesions had a significantly higher volume and number compared to CVS− lesions in all the four brain regions analyzed [deep/subcortical WM, periventricular, juxtacortical, and infratentorial; (*p* < 0.001 for all, both for volume and number), Table 2].

**Table 2:** Volume and topography of CVS+ and CVS− lesions in the whole cohort and according to age groups.

	**CVS+**	**CVS−**	***p*-value**
Total lesions, *n* (%)	3,645 (68.7)	1,658 (31.3)	–
**Whole cohort, Lesion volume (mm** ^3^ **), median (** ***range*** **)**			
Periventricular	1,292 (26–7,969)	224 (17–1,713)	**< 0.001**
Infratentorial	296 (110–555)	45 (13–88)	**< 0.001**
Juxtacortical	161 (75–273)	34 (16–54)	**< 0.001**
Deep/subcortical WM	283 (72–526)	74 (29–255)	**< 0.001**
	596 (158–1,229)	141 (56–270)	**< 0.001**
**Whole cohort, Lesion location**, ***n*** **(*****%*****)**			
Periventricular	584 (80)	146 (20)	**< 0.001**
Infratentorial	527 (85.6)	89 (14.4)	**< 0.001**
Juxtacortical	640 (61.3)	404 (38.7)	**< 0.001**
Deep/subcortical WM	1894 (65.1)	1019 (34.9)	**< 0.001**
**Age Group 1 (18–30), Lesion location**, ***n*** **(%)**			
Periventricular	136 (84.5)	25 (15.5)	**< 0.001**
Infratentorial	112 (85.5)	19 (14.5)	**< 0.001**
Juxtacortical	171 (67.6)	82 (32.4)	**< 0.001**
Deep/subcortical WM	411 (72.1)	159 (27.9)	**< 0.001**
**Age Group 2 (31–44), Lesion location**, ***n*** **(%)**			
Periventricular	178 (84)	34 (16)	**< 0.001**
Infratentorial	161 (88.5)	21 (11.5)	**< 0.001**
Juxtacortical	187 (64.9)	101 (35.1)	**< 0.001**
Deep/subcortical WM	570 (73.4)	207 (26.6)	**< 0.001**
**Age Group 3 (45–55), Lesion location**, ***n*** **(%)**			
Periventricular	123 (75.9)	39 (24.1)	**< 0.001**
Infratentorial	113 (85)	20 (15)	**< 0.001**
Juxtacortical	161 (59.2)	111 (40.8)	**0.033**
Deep/subcortical WM	558 (67.1)	273 (32.9)	**< 0.001**
**Age Group 4 (56–77), Lesion location**, ***n*** **(%)**			
Periventricular	147 (75.4)	48 (24.6)	**< 0.001**
Infratentorial	141 (82.9)	29 (17,1)	**< 0.001**
Juxtacortical	121 (52.4)	110 (47.6)	0.19
Deep/subcortical WM	355 (48.3)	380 (51.7)	0.085

CVS, Central Vein Sign.

### CVS proportion-based diagnostic thresholds vs. simplified algorithms

Based on the 35% and the 40% of CVS proportion-based diagnostic thresholds (29–31), 119 of the 120 included patients were perivenular positive for both thresholds. In one patient, %CVS+ lesions was 28% (age Group 4, secondary progressive phenotype, and history of migraine); in the other patient, it was 39% (age Group 2, secondary progressive phenotype, smoke, and migraine). When applying the simplified algorithms, 6-lesion (8) and 3-lesion rules (32), 119 and 111 of the 120 included patients were perivenular positive, respectively.

### CVS relationship with MS phenotype, RFs for SVD, and migraine

Patients with relapsing-remitting multiple sclerosis showed a higher percentage of CVS+ lesions compared to patients with PMS (76.9%, range 40–100 vs. 67.3%, range 27.7–100%; *p* = 0.002).

The median percentage of CVS+ lesions decreased from age Group 1 to age Group 4 (for age Group 1: median 79.7%, range 60.3–100%; for age Group 2: median 79.1%, range 39.1–100%; for age Group 3: median 71.8%, range 40–100%; for age Group 4: median 57.7%, range 27.7–100%). Differences in the median percentage of CVS+ lesions were observed among all age groups, except for age Group 2 vs. age Group 3 (Table 3).

**Table 3:** CVS+ lesions percentage comparisons among age groups, MS phenotype, RFs for SVD, and migraine.

	**CVS+ (% lesions), median (range)**	**p-value**
**Age**		
Age Group 1: 18–30 (*n* = 30)^1^	79.7 (60.3–100)	1 vs 2 ***p*** **=** **0.026**
Age Group 2: 31–44 (*n* = 30)^2^	79.1 (39.1–100)	1 vs 3 ***p*** **<** **0.001** 2 vs 3 0.088
Age Group 3: 45–55 (*n* = 30)^3^	71.8 (40–100)	1 vs 4 ***p*** **<** **0.001**
Age Group 4: 56–77 (*n* = 30)^4^	57.7 (27.7–100)	2 vs 4 ***p*** **<** **0.001**
		3 vs 4 ***p*** **=** **0.017**
**MS type**		
RR (*n* = 84)	76.9 (40–100)	**0.002**
PMS (*n* = 36)	67.3 (27.7–100)	
**HT**		
No (*n* = 103)	74.7 (27.7–100)	**0.031**
Yes (*n* = 17)	61.9 (43.3–100)	
**Diabetes or glucose intolerance**		
No (*n* = 118)	74.0 (27.7–100)	0.33
Yes (*n* = 2)	60.8 (49.2–72)	
**Smoke**		
No (*n* = 57)	72.4 (27.7–100)	0.84
Yes (*n* = 63)	75 (39.1–100)	
**BMI**		
18–24.9 (*n* = 79)	75 (27.7–100)	0.27
≥25 (*n* = 41)	72 (40–100)	
**Hypercholesterolemia**		
No (*n* = 99)	74.7 (27.7–100)	0.07
Yes (*n* = 21)	68 (43.1–100)	
**Cumulative RFs number**		
0 (*n* = 31)	74.7 (40–100)	0.55
1 (*n* = 52)	77.1 (27.7–100)	
2 (*n* = 20)	65.8 (39.1–93.3)	
3–4 (*n* = 17)	66.7 (43.5–88.1)	
**Migraine (with or without aura)**		
No (*n* = 86)	76.4 (40–100)	**0.032**
Yes (*n* = 34)	65.8 (27.7–100)	

CVS, Central Vein Sign.

**Figure 1:**
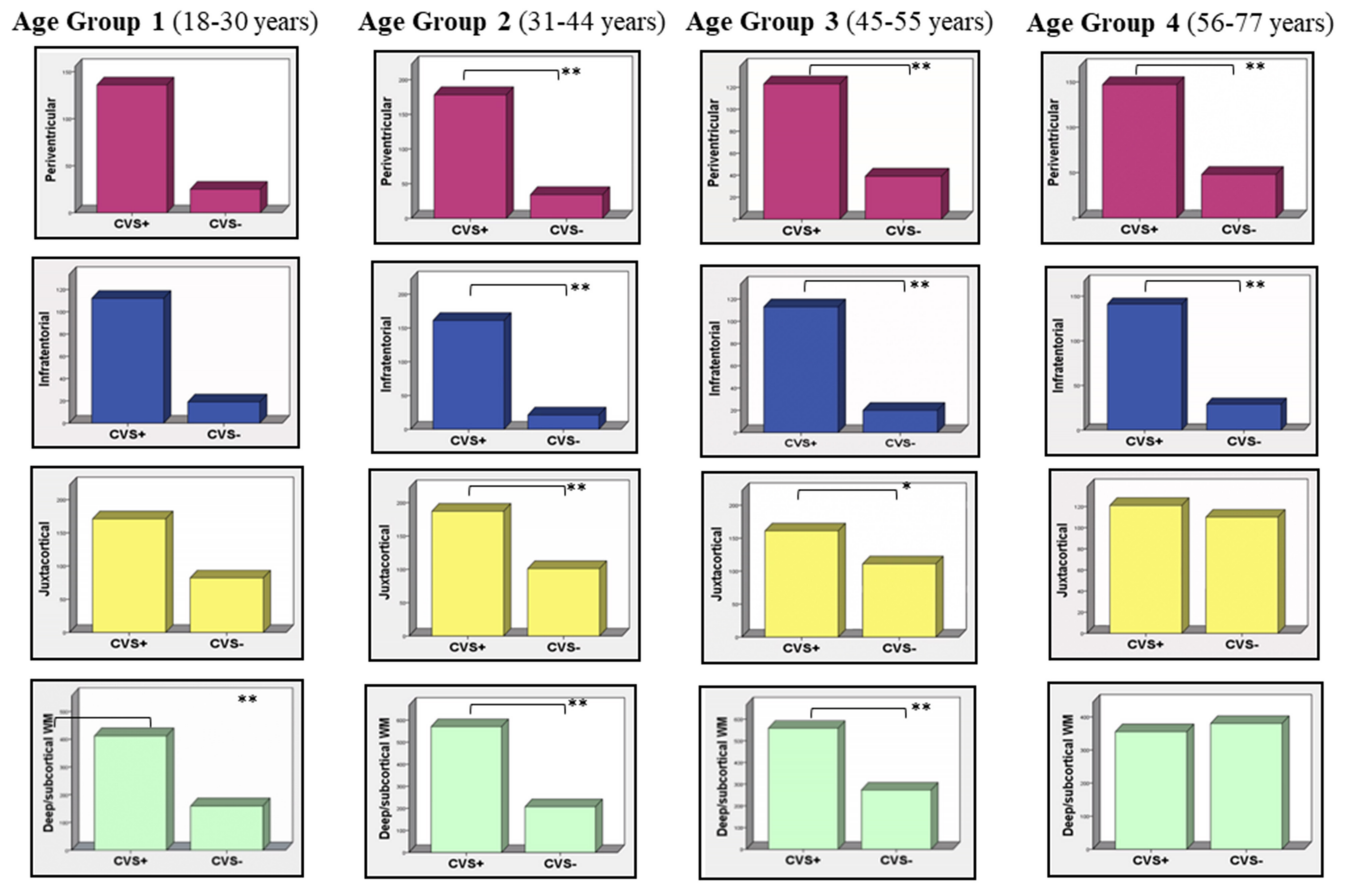
CVS+ and CVS− distribution according to age groups and brain subregions. In all age groups and brain subregions, CVS+ lesion number is higher than CVS− lesions (**p < 0.001; *p = 0.033), except for the juxtacortical area in age Group 4 where the difference is not significant. Note that in the deep/subcortical WM in age Group 4 (57–77 years) CVS− lesion number rises and becomes higher than CVS+ lesions, although not statistically significant. CVS, Central Vein Sign.

When patients with MS were stratified according to age groups, we found that, in all age groups and brain subregions, CVS+ lesion number was higher than CVS− lesions [*p* < 0.001 for all, except for (i) juxtacortical area in age Group 3 (*p* = 0.033) and (ii) juxtacortical area in age Group 4 where the difference was not significant], excluding the deep/subcortical WM in age Group 4, where CVS− lesion number was higher than CVS+ lesions, although not reaching statistical significance (Table 2, Figure 1).

Patients with HT showed a lower percentage of CVS+ lesions (median: 61.9%, range 43.3–100%) compared to patients not diagnosed with HT (median: 74.7%, range 27.7–100%; *p* = 0.031). Patients with migraine had a lower percentage of CVS+ lesions (median: 65.8%, range 27.7–100%) compared to patients without migraine (median: 76.4%, range 40–100%; *p* = 0.032). A trend was observed between patients with hypercholesterolemia and no hypercholesterolemia (median: 68%, range 43.1–100% vs median 74.7%, range 27.7–100% respectively; *p* = 0.078). For the variables: smoking/no smking, BMI ≥ 25/BMI ≤ 25, diabetes or glucose intolerance/ no diabetes or glucose intolerance, and cumulative number of RFs for SVD, no differences in terms of CVS+ vs CVS− lesion median percentage were observed in terms of CVS+ vs CVS− lesion median percentage (Table 3).

**Figure 2:**
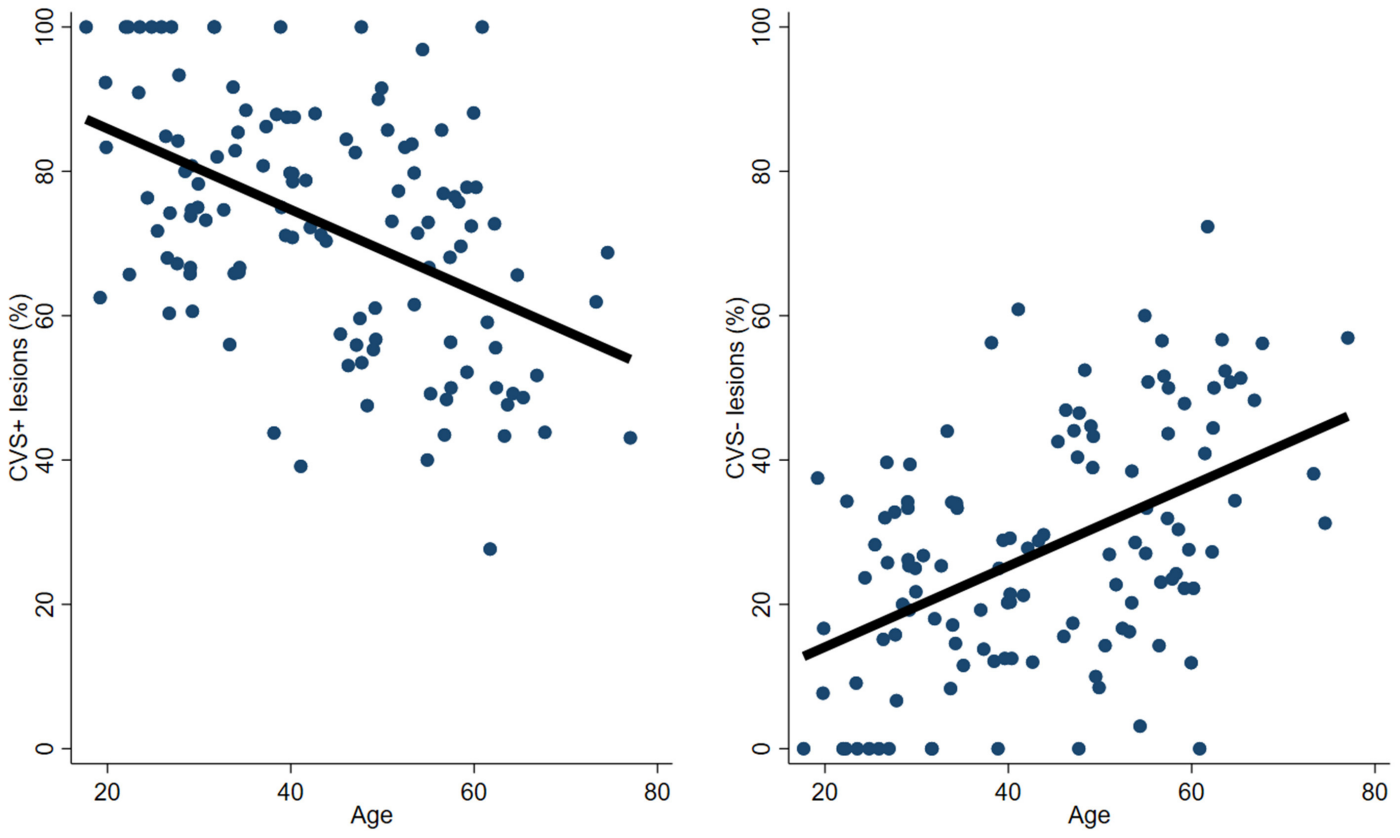
Association between patient's age and the frequency of CVS+ and CVS− lesions. An inverse correlation was found between %CVS+ lesions and age (*r* = −0.46; *p* < 0.001), while a positive correlation was found between %CVS− lesions and age (*r* = 0.46; *p* < 0.001). CVS, Central Vein Sign.

A negative correlation was found between %CVS+ lesions and age (*r* = −0.46; *p* < 0.001, Figure 2) and between %CVS+ lesions and disease duration (*r* = −0.24; *p* = 0.008), while a trend was observed with BMI (*r* = −0.17; *p* = 0.058).

In the multivariable model, including age, migraine, the cumulative number of RFs for SVD, HT, MS phenotype, and disease duration, age and migraine were independently associated with the %CVS+ lesions (model R^2^ 0.25; *p* < 0.001 for age and *p* = 0.013 for migraine).

### Microstructural features of CVS+ and CVS− lesions evaluated by the SMT diffusion model

Compared to CVS− lesions, CVS+ lesions showed higher EXTRAMD (*p* = 0.001), higher EXTRATRANS (*p* = 0.001), and lower INTRA (*p* = 0.02). In the deep/subcortical WM, juxtacortical, and infratentorial areas, EXTRAMD was higher in CVS+ lesions compared to CVS− lesions (*p* = 0.001, 0.01, and 0.05, respectively), while in the periventricular region, we observed the opposite result (*p* = 0.001). In the deep/subcortical WM and infratentorial areas, EXTRATRANS was higher in CVS+ lesions compared to CVS− lesions (*p* = 0.001 and *p* = 0.04, respectively), while in periventricular and juxtacortical regions, no differences were observed. In the deep/subcortical WM and periventricular areas, INTRA was lower in CVS+ lesions compared to CVS− lesions (*p* = 0.001 for both), while in infratentorial and juxtacortical regions, no differences were observed (Table 4).

**Table 4:** SMT metrics comparisons between CVS+ and CVS− lesions.

	**CVS+**	**CVS−**	*p*-value^*^	***p*-value adjusted for m.c.^∧^**
**EXTRAMD (inflammation), mean (** ***SD*** **) mm** ^**2**^ **/s**	0.00144 (0.000245)	0.00139 (0.000228)	**< 0.001**	**0.001**
Deep/subcortical WM	0.00139 (0.000128)	0.00135 (0.000114)	**< 0.001**	**0.001**
Periventricular	0.00154 (0.000226)	0.00163 (0.000301)	**< 0.001**	**0.001**
Juxta	0.00132 (0.000175)	0.00129 (0.000190)	**0.007**	**0.01**
Infratentorial	0.00141 (0.000251)	0.00134 (0.000260)	**0.035**	**0.05**
**EXTRATRANS (demyelination), mean (** ***SD*** **) mm** ^**2**^ **/s**	0.00117 (0.000288)	0.00111 (0.000252)	**< 0.001**	**0.001**
Deep/subcortical WM	0.00109 (0.000182)	0.00105 (0.000145)	**< 0.001**	**0.001**
Periventricular	0.00131 (0.000252)	0.00135 (0.000333)	0.13	0.15
Juxta	0.00114 (0.000176)	0.00112 (0.000194)	0.08	0.11
Infratentorial	0.00103 (0.000293)	0.000952 (0.000296)	**0.027**	**0.04**
**INTRA (fiber disruption), mean (** ***SD*** **)**	0.399 (0.129)	0.409 (0.124)	**0.012**	**0.02**
Deep/subcortical WM	0.425 (0.113)	0.449 (0.0976)	**< 0.001**	**0.001**
Periventricular	0.341 (0.0969)	0.378 (0.109)	**< 0.001**	**0.001**
Juxta	0.309 (0.0932)	0.301 (0.0894)	0.17	0.19
Infratentorial	0.507 (0.122)	0.530 (0.138)	0.11	0.14

m.c., multiple comparisons.

^*^P-value obtained from the GEE model and adjusted for age, MS phenotype, and gender; ^∧^Adjustment for multiple comparisons using the false-discovery rate approach. CVS, Central Vein Sign; SMT, Spherical Mean Technique.

**Figure 3:**
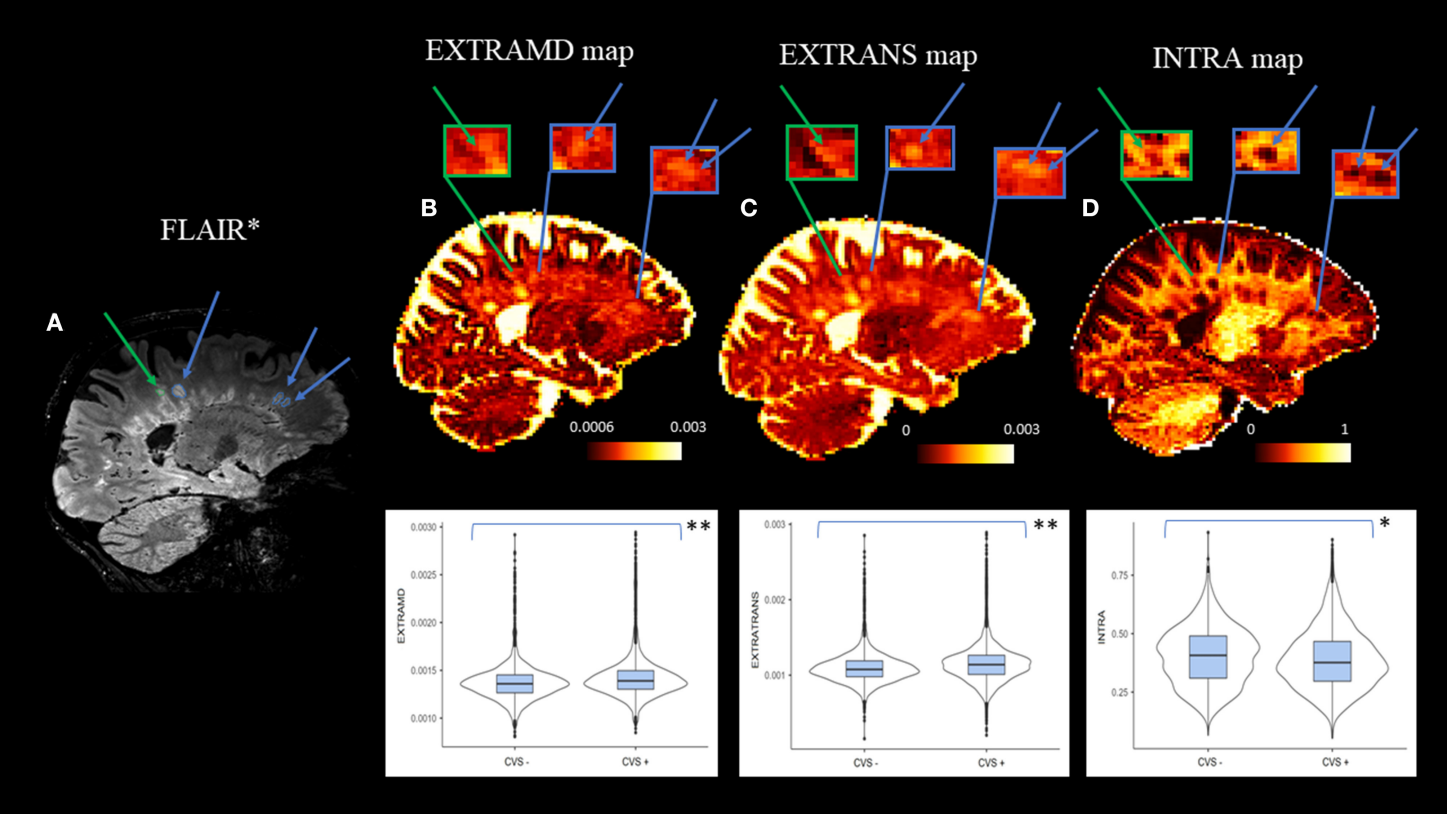
Selected sagittal FLAIR* **(A)** and SMT-derived EXTRAMD **(B)**, EXTRATRANS **(C)** and INTRA **(D)** maps of a 62-year-old patient diagnosed with multiple sclerosis. At the top: the color bar expresses each SMT-metric: adimensional unit for INTRA (intraneurite signal fraction, corresponding to fiber disruption), mm^2^/sec for EXTRAMD (extraneurite mean diffusivity, corresponding to inflammation severity), and EXTRATRANS (extraneurite transverse diffusivity, corresponding to the degree of demyelination) measures. Arrows and zoomed-in boxes indicate the presence of representative CVS+ lesions (blu arrows) and CVS− lesion (green arrows) to highlight the differences in SMT metrics among them. At the bottom: violin plots showed higher inflammation, demyelination, and fiber disruption in CVS+ lesions compared to CVS− lesions (**p = 0.001; *p = 0.02). CVS, Central Vein Sign.

SMT-metrics maps within representative CVS+ and CVS−lesions (at the top) and their graphical representation by violin plots (at the bottom) are shown in Figure 3.

## EDSS scores correlations with SMT metrics

The EDSS scores were correlated with SMT metrics extracted from CVS+ and CVS− lesions but no significant results were observed, while a significant negative correlation was detected between SMT-intra of the NAWM and EDSS (*p* = 0.014 *r* = −0.226).

## Discussion

In this study, we investigated the impact of RFs for SVD and migraine on the global and subregional brain CVS assessment in a large cohort of patients with MS stratified according to age and thus applied the SMT diffusion model to evaluate whether perivenular lesions show distinctive microstructural features compared to non-perivenular lesions. We focused on the different risk factors for SVD (age, BMI, smoking, HT, diabetes or glucose intolerance, hypercholesterolemia) and migraine, due to their high prevalence in the common population, including patients with MS (13, 44). Unlike MS, histopathological studies in SVD revealed that the anatomical target of tissue damage is mostly represented by the arteriolar side of vascular microcirculation (19, 45), where vessel lumen restriction and chronic hypoperfusion mainly occur. Although the pathophysiology of migraine-related deep WM hyperintensities is poorly understood, both ischemic and inflammatory mechanisms have been proposed, as there is increased cerebral vulnerability to ischemia in migraineurs, as well as evidence of BBB disruption during migraine attacks (18).

Among the RFs for SVD, age was the strongest inverse predictor of the percentage of CVS+ lesions, while HT, although associated with a higher prevalence of CVS− lesions, did not survive as a significant predictor in the regression analysis. The low percentage of MS patients with HT in our sample (as for patients with diabetes, higher BMI, and smokers) may explain these findings. One of the most novel aspects of our study was the investigation of migraine impact on the percentage of CVS+ lesions. MS patients with migraine showed a higher percentage of CVS− lesions compared to MS patients without migraine. Furthermore, migraine also survived as an inverse predictor of the percentage of perivenular lesions in the regression analysis. Interestingly, analyzing the demographic and clinical features of patients with MS who did not fulfill the 40% thresholds approach in our sample, we observed that both MS patients had suffered or suffered from migraine. Although our data confirmed that the previously proposed CVS proportion-based thresholds (29–31) remain valid for differential diagnosis, they may suggest that migraine, as well as aging, could be able to affect CVS performance and, thus, it should be carefully considered in the radiological workflow of patients with high clinical suspicion of MS.

Furthermore, in order to investigate whether older age has a more preferential impact on the CVS assessment in some brain subregions than in others, we considered the distribution of the CVS+ and CVS− lesions in brain areas considered specific (periventricular, infratentorial, and juxtacortical) and not specific (deep/subcortical WM) for MS. CVS+ lesion volume was higher than CVS–lesion volume, both considering the global brain and the four subregions analyzed, where CVS+ lesions were also numerically prevalent. In a recent study (12), it was reported that CVS+ lesion volume in the whole brain and CVS+ lesion number in the deep/subcortical WM were higher than CVS− lesion volume and CVS− lesion number in the global brain and deep/subcortical WM, respectively, although there was no statistical significance between them. The larger sample size and the higher number of lesions analyzed in our study may partially explain these different findings. Nevertheless, conflicting results emerged also in the juxtacortical area, where Guisset et al. (12) found that CVS− lesions were numerically prevalent compared to CVS+ lesions. CVS evaluation in the juxtacortical area may be challenging due to the possible effect of distortion artifacts intrinsic to EPI-T2^*^ images. To improve the detection rate of CVS+ lesions, we decided to perform EPI-T2^*^ images after contrast agent administration, following the suggestion of previous studies (46–48). It is possible that T1 shortening, due to gadolinium administration, may lead to an increase in the phase effects around blood vessels, thus improving the visibility of the central vein (47, 49). In our study, the CVS assessment in gadolinium enhanced susceptibility images could have helped to optimize the detection of perivenular lesions on the whole brain but also in challenging areas.

After having stratified MS patients according to age to evaluate the CVS in the different brain subregion, we found that in brain subregions considered typical of MS (periventricular, infratentorial, and juxtacortical), the relationship between CVS+/CVS− lesion number showed a clear prevalence of CVS+ on CVS− lesions in all age groups, except for juxtacortical areas in the 56–77 years group. An overestimation of CVS− lesions in the juxtacortical area throughout all age groups due to the abovementioned technical issues, despite our attempt to improve CVS detection by acquiring EPI-T2^*^ images after contrast injection, may partially explain our findings. Furthermore, despite both SVD and migraine-related WM T2-weighted hyperintensities being mostly located in the deep/subcortical WM, different studies showed that juxtacortical areas may also be involved (50, 51). Interestingly, we found that in age Group 4 (56–77 years) CVS− lesion number increased to become higher than CVS+ lesions in the deep/subcortical WM, although no statistical significance was found.

Therefore, driven by our findings about the impact of age and migraine on the percentage of CVS+ lesions and the inversion of CVS+/CVS− lesions prevalence in deep/subcortical WM in older patients with MS, we decided to use the SMT model to investigate the pathological substrate of CVS+ and CVS− lesions. The choice to use SMT relied on its interesting basic assumptions and its encouraging recent results in MS (20, 23, 43). Overcoming the issue represented by the fixed intrinsic diffusivity of other multicompartment models (20), SMT considers WM as a two-compartment (intra- and extra-axonal) tissue and provides signal fraction and diffusion metrics per axon without confounds from fiber direction, crossing, or dispersion (43). Histopathologic validation of SMT has been performed in the animal model of tuberous sclerosis, where the absence of neuroinflammation makes the detection of CNS axonal injury and demyelination more suitable. Thus, although obtained with a different disease model, the provided validation against axonal histology is fundamental. It applies to any condition affecting myelin and axonal integrity and supports the ability of SMT to quantify axonal content without artifactual effects from fiber-crossing and orientation dispersion (18). This is particularly important in MS because many WM voxels contain complex fiber configurations, and fiber arrangements widely vary within MS lesions. Thus, these fiber orientation-independent diffusion metrics may provide more accurate estimates of axon integrity. SMT has been already applied in different *in vivo* studies focusing on the brain (22) and spinal cord (23) of patients with MS, demonstrating to be helpful in differentiating MS lesions damage from the NAWM as well as the NAWM of patients with MS from that of healthy controls (20) and in characterizing pathological features within MS lesions (52). Furthermore, it has been demonstrated that DTI, neurite orientation dispersion and density imaging (NODDI), and SMT concur on the direction of tissue changes in MS, providing consistent descriptors of tissue microstructure useful in monitoring MS in clinical trials and practice (53). In this study, we demonstrated that SMT was able to investigate the pathological substrates of CVS+ and CVS− lesions and detect distinctive features capable of differentiating them from each other. Compared to CVS− lesions, perivenular lesions showed higher EXTRAMD, indirectly reflecting higher free water content, higher EXTRATRANS, indirect expression of a decrease in myelin content, and lower INTRA, suggestive of a higher degree of axonal damage and fiber disruption. Thus, we could suggest that perivenular lesions, typical of MS, were characterized by a more severe degree of inflammation, demyelination, and fiber disruption than non-perivenular lesions, possibly associated with different pathophysiological mechanisms. Similar strong evidence was found comparing all SMT metrics within CVS+ and CVS− lesions clustered in the deep/subcortical WM. Fiber disruption seemed to also be higher in perivenular lesions located in periventricular areas, while cerebrospinal fluid (CSF) contamination could have affected extraneurite compartment metrics (EXTRAMD >in CVS− lesions; no difference was found between CVS+ and CVS− lesions in EXTRATRANS). Similar, although weaker, differences were found in juxtacortical and infratentorial areas. Indeed, compared to CVS− lesions, a higher inflammatory component was detected in CVS+ lesions located in both regions and a more pronounced degree of demyelination was found in infratentorial CVS+ lesions. Technical issues may have affected these findings. Because the voxel signal is a sum of all tissue signals within the voxel, finite image resolution inevitably causes a mixture of signals at the interface of two tissues. This phenomenon, known as partial volume effect (PVE), may obscure small lesions near the interface between tissues (54) and, quantitatively, may cause errors in volumetric measurements using structural MRI or region-of-interest (ROI) measurements using diffusion-weighted imaging (55). Thus, this limitation in SMT metrics extraction cannot be disregarded in periventricular and juxtacortical areas, where CSF contamination and the different tissue cytoarchitecture of gray matter characterize the corresponding WM interfaces. Furthermore, the lower mean volume of juxtacortical and infratentorial T2-weighted hyperintensities compared to the deep/subcortical WM may also contribute to explain why SMT metrics seem to perform worse in differentiating perivenular from non-perivenular lesions in these areas.

Finally, we found a correlation between EDSS score as a clinical parameter and SMT metrics extracted from the NAWM but not between EDSS scores and SMT metrics obtained from CVS+ and CVS− lesions. The pathological, and, thus, microstructural, heterogeneity of FLAIR hyperintense lesions, ranging from early lesions to T1-hypointense “black holes” and the exclusion of a considerable amount of FLAIR lesions from the CVS assessment, whose SMT metrics were thus not extracted in our study, might explain our findings. Conversely, the correlation we found between the NAWM microstructural damage and the ESSS is in line with that shown in a recent paper (53), suggesting that the greater the widespread axonal damage, the poorer the clinical status. These findings may indicate initial hints about the clinical potential of SMT diffusion derived metrics in explaining disability in MS *in vivo*.

This study is not without limitations. First, the presence of a comparison group including non-MS patients suffering from RFs for SVD and/or migraine would have been very helpful to investigate whether CVS− lesions in MS and non-MS patients possibly share microstructural features, thus potentially contributing to validating our findings. Moreover, the cross-sectional design of this study does not allow us to evaluate how and where the new T2-weighted hyperintensities develop over time and their temporal relationships with aging and other comorbidities in patients with MS. The relatively low incidence of MS patients with RFs for SVD in our sample may have underestimated the role of HT, above all, in reducing the percentage of CVS+ lesions and thus affecting CVS performance. Finally, we did not include other potential causes of WM lesions, such as a macroangiopathic disease, i.e., lacunar infarcts-, hemodynamic changes, and abnormalities of heart rhythm (i.e., atrial fibrillation) or structure (i.e., patent foramen ovale), that could increase the prevalence of CVS− lesions within the brains of patients with MS.

In conclusion, this study demonstrated that aging has a relevant impact on reducing the percentage of CVS+ lesions in patients with MS. This effect is already clear when the whole brain is considered but becomes even more evident when the deep/subcortical WM, a region not typical of MS, is specifically analyzed. Indeed, in this site, non-perivenular lesions become more prevalent than perivenular lesions in older patients with MS. Although the use of three periventricular lesions instead of 1, as required by current MS criteria for DIS (24), surely helps in reducing the risk of misdiagnosis or wrong interpretation of disease activity in older and comorbid patients with MS (56), it lacks a pathophysiological basis. Furthermore, vascular leukoaraiosis is typically extended around ventricles and its differentiation from confluent MS lesions may be very challenging. Our findings suggest that thanks to the use of MRI biomarkers closely linked to MS pathophysiology -as the CVS-, also “non-DIS” regions may become very informative and help to prevent diagnostic misinterpretation.

Among the other comorbidities, for the first time, we showed that migraine may also play a significant role in increasing the amount of non-perivenular lesions in younger patients with MS. Furthermore, we demonstrated that SMT-derived metrics may provide a deep characterization of microstructural features within WM lesions and, for the first time, that these metrics seem to be able to differentiate perivenular lesions, characterized by higher levels of inflammation, demyelination, and fiber disruption, from non-perivenular lesions, for which other pathophysiological mechanisms could be suggested.

Therefore, in our opinion, the development of a new non-perivenular T2-weighted hyperintensity, especially if located in the deep/subcortical WM in older patients with MS, should be considered a “red flag” for a pathophysiology other than MS disease activity. A careful evaluation of comorbidities during CVS assessment for the diagnosis and monitoring of MS should be mandatory, to avoid misleading interpretations and potentially inappropriate therapeutic strategies.

## Data availability statement

The raw data supporting the conclusions of this article will be made available by the authors, without undue reservation.

## Ethics statement

The studies involving human participants were reviewed and approved by Local Ethic Committee of the IRCCS Ospedale Policlinico San Martino (Genoa). The patients/participants provided their written informed consent to participate in this study.

## Author contributions

CL: designed and conceptualized study, major role in the acquisition and analysis of data, and drafted the manuscript for intellectual content. FT: major role in the acquisition and analysis of data and revised the manuscript for intellectual content. SR and AS: major role in the analysis of data. GB and ES: major role in the acquisition of data and revised the manuscript for intellectual content. NB, EM, and NM: major role in the acquisition of data. LR: major role in the acquisition of data and study conceptualization and revised the manuscript for intellectual content. MC: revised the manuscript for intellectual content. SS: major role in the analysis of data and revised the manuscript for intellectual content. MI: design and conceptualized study, analyzed the data, and revised the manuscript for intellectual content.
